# Economics and health economics as a major determinant towards HiAP

**DOI:** 10.1093/eurpub/ckac129.578

**Published:** 2022-10-25

**Authors:** J Santos

**Affiliations:** 1 EUPHA-ECO; 2 MEDCIDS – Department of Community Medicine, University of Porto, Porto, Portugal; 3 CINTESIS, Center for Health Technology and Services Research, Porto, Portugal

## Abstract

COVID-19 pandemic response was an opportunity to advocate for HiAP as everyone developed opinions on how policies could affect the different dimensions, including health and economy. Health planning, from the local to the international level, is the main setting where we still need to advocate for the inclusion of all political sectors and actors in order to ensure an effective HiAP implementation. From a more technical perspective, there is still a lot to do when it comes to implementing and improving economic evaluations, including on data on costs, valuing benefits or using cost-effectiveness approaches.

**Speakers/Panellists:**

**Nikhil Gokani**

EUPHA-LAW

School of Law, University of Essex, Essex, UK

